# Rapidly Measuring Scattered Polarization Parameters of the Individual Suspended Particle with Continuously Large Angular Range

**DOI:** 10.3390/bios12050321

**Published:** 2022-05-10

**Authors:** Yan Chen, Hongjian Wang, Ran Liao, Hening Li, Yihao Wang, Hu Zhou, Jiajin Li, Tongyu Huang, Xu Zhang, Hui Ma

**Affiliations:** 1 School of Physics and Optoelectronic Engineering, Yangtze University, Jingzhou 434023, China; 201971160@yangtzeu.edu.cn (Y.C.); 201971151@yangtzeu.edu.cn (H.Z.); 2 Shenzhen International Graduate School, Tsinghua University, Shenzhen 518055, China; whj20@mails.tsinghua.edu.cn (H.W.); lhn19@mails.tsinghua.edu.cn (H.L.); yihao-wa20@mails.tsinghua.edu.cn (Y.W.); lijiajin21@mails.tsinghua.edu.cn (J.L.); hty19@mails.tsinghua.edu.cn (T.H.); mahui@tsinghua.edu.cn (H.M.); 3 College of Chemistry and Environmental Engineering, Yangtze University, Jingzhou 434020, China; zhangxu@yangtzeu.edu.cn

**Keywords:** optical scattering, polarization parameters, continuously large angular range, individual suspended particle

## Abstract

Suspended particles play a vital role in aquatic environments. We propose a method to rapidly measure the scattered polarization parameters of individual suspended particles with continuously large angular range (PCLAR), from 60° to 120° in one shot. A conceptual setup is built to measure PCLAR with 20 kHz; to verify the setup, 10 μm-diameter silica microspheres suspended in water, whose PCLAR are consistent with those simulated by Mie theory, are measured. PCLAR of 6 categories of particles are measured, which enables high-accuracy classification with the help of a convolutional neural network algorithm. PCLAR of different mixtures of *Cyclotella stelligera* and silica microspheres are measured to successfully identify particulate components. Furthermore, classification ability comparisons of different angular-selection strategies show that PCLAR enables the best classification beyond the single angle, discrete angles and small-ranged angles. Simulated PCLAR of particles with different size, refractive index, and structure show explicit discriminations between them. Inversely, the measured PCLAR are able to estimate the effective size and refractive index of individual *Cyclotella* cells. Results demonstrate the method’s power, which intrinsically takes the advantage of the optical polarization and the angular coverage. Future prototypes based on this concept would be a promising biosensor for particles in environmental monitoring.

## 1. Introduction

Suspended particles can be divided into three major categories, including microalgae, microplastics, and sediments [1], which are important indicators for the health status of the aquatic environment. Monitoring the categories and physiological states of microalgae can prevent toxic blooms and help understand the changes of carbon cycle [2]. The emerging pollutant, microplastics, threatens both marine organisms and human beings [3]. The sediments are important components of the aquatic environment, which easily adsorb the pollutant matter, and affect the underwater propagation of sunlight [4]. However, the rapid and in situ classification of these suspended particles is still challenging for the community.

Optical microscopy is a common method to probe the suspended particles by pre-treatment and specialized skills [5,6], which nowadays is assisted by new techniques such as computer-assistant recognition and multimodality measurement [7,8]. Recently, some other imaging tools have been developed, showing a great potential in phytoplankton taxonomy [9,10], but all these imaging methods are still limited by the tradeoff between the acquisition speed, image resolution, and visual filed [11].

Meanwhile, the acoustic backscatter profiling sensor and turbidimeter are two popular techniques in bulk water analysis, and their results show a good correlation with the concentration of the target particles [12,13]. However, these methods can only be used to probe the particles of the bulk volume, which indicate that they may easily meet their bottleneck in further detailed analysis of different components [14].

Scattering is related to the physical and optical properties of the particles, such as size, shape, and the refractive index [15,16]. The scattering measurement has been applied in diverse fields, such as oceanic science, astrophysics, meteorology, and material research [17]. Compared with traditional scattering measurement, polarized light scattering measurement can provide more physical information of particles [18], and the polarization state of light can be used to quantitatively evaluate the biogenic particles in water [19]. Recently, by taking advantage of individual particle measurement, the classification of individual microalgae and microplastics with the scattered light at a backward continuous angle of 120° was reported [20]. Because polarized light scattering at 120° is sensitive to the intracellular structural changes of cells, this shows its potential for environmental monitoring, such as early warning of the toxic blooms [21,22]. Additionally, the statistical Mueller matrix is introduced to enhance the discrimination ability [23]. Meanwhile, the polarized light scattering at several discrete angles, such as 60°, 85°, and 115°, was proposed to discriminate different categories of aerosols [24,25]. However, these scattering measurements based on the single angle, or the discrete angles, easily meet their bottlenecks during the classification of particles. To measure the continuous scattered light, angularly resolved scattering measurement is proposed and realized by analyzing the two-dimensional angular optical scattering patterns to characterize particles [26]; however, this kind of experimental setup is subject to the acquisition speed and the sensitivity of the camera, and the intensity-only measurement also limits its classification ability for particles.

In this work, by using a polarization line scan CCD, an experimental setup is built based on the polarized light scattering to rapidly measure the polarization parameters of individual suspended particles with continuously large angular range (PCLAR). Firstly, the optical polarization measurement is calibrated, and the suspension of the 10 μm diameter silica microspheres is used to validate the PCLAR and their angular range by comparing them with the simulation result of Mie theory. Different categories of particle are separately measured with the setup, and the measured PCLAR are used by the convolutional neural network (CNN) algorithm to build a classifier that can well classify these categories of particles. Then, the experiments are conducted for the mixture of the particles, and the classifier is used to successfully identify the particulate components in mixtures. The classification abilities of different angular selection strategies are compared, and PCLAR with the continuously large angular range performs best. The PCLAR of particles with different size, refractive index, and structures are simulated, which show the explicit discriminations between these particles. Inversely, PCLAR are used to estimate the effective size and refractive index of the individual *Cyclotella* cells. In this work, our results demonstrate the power of our method and show the intrinsic advantage of the scattered polarization parameters within a continuously large angular range in probing the suspended particles. With these advantages, future prototypes based on this concept would be a promising biosensor to probe and monitor the suspended particles in aquatic environments.

## 2. Materials and Methods

### 2.1. Samples

Different particles are measured in this work, including 3 categories of microplastics with different sizes and structures, i.e., polystyrene microsphere with a diameter of 5 μm (PS5), polystyrene microsphere with a diameter of 10 μm (PS10), polystyrene microsphere with a diameter of 10 μm and uniformed holes on its surface (PSH), one category of sediment, i.e., 10 μm-diameter silica microsphere (SiO_2_), and two categories of microalgae cells, *Euglena gracilis* (EU) and *Cyclotella stelligera* (CY).

During the preparation of the suspensions, first, the powders of PS5, PS10, PSH, and SiO_2_ are separately dispersed in distilled water as their own concentrates whose concentrations are 8.01 $\times \;10^{3}$, 1.32 $\times \;10^{3}$, 1.04 $\times \;10^{3}$, and 5.40 $\times \;10^{2}$ particles per milliliter (mL), respectively. For microalgae, we sample 400 μL of their concentrates in 5 mL distilled water in the sample pool, and these are measured by the setup. All of the microalgae were taken at their logarithmic growth state and provided by INFORE ENVIRO Co., Ltd. (Foshan, China), and other non-biological microsphere were provided by Suzhou Nanomicro Technology Co., Ltd. (Suzhou, China).

### 2.2. Experimental Setup

**Figure 1:**
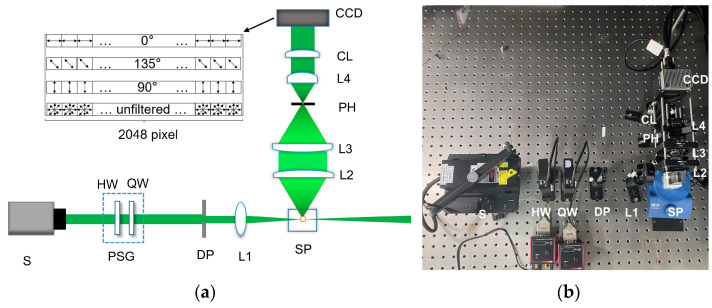
(**a**) Schematic of experimental setup: S, light source; PSG, polarization state generator; HW, half-wave plate; QW, quarter-wave plate; DP, diaphragm; L1, L2, L3, L4, lens; SP, sample pool; PH, pinhole; CL, cylindrical lens. Inserted image in the dashed box: pixel arrangement of polarization line scan CCD; (**b**) Physical picture of the built experiment setup.

The schematic diagram of the experimental setup is shown in Figure 1a; light with 532 nm wavelength is emitted from the light source (S). Then the polarization state of light is modulated by the polarization state generator (PSG), which consists of a rotating half-wave plate (HW) and a rotating quarter-wave plate (QW). In this work, we use PSG to set the illuminating light as the fixed 45° linearly polarized, then the diaphragm (DP) and the lens (L1) are used to focus the polarized light into a very small light spot in the sample pool (SP). In SP, the particles are suspended in water with an electromagnetic stirrer rotating at a speed of 150 rounds per minute (rpm). Once the particle is illuminated by the light spot, the scattered light can be detected by the receiving optical path. After the light is received and collimated by lens L2, the collimated light is focused by lens L3. Both L2 and L3 share the same focal length of 30 mm and diameter of 2 inches.

Subsequently, the focused light passes through a pinhole (PH), and is then collimated by the lens L4 with 15 mm focal length to change the diameter of the light beam into 1 inch. Then, a cylindrical lens (CL) is applied to compress the circular light beam into a light sheet, to raise the intensity density. Finally, the light sheet is recorded at one shot by the polarization line scan CCD (P4-CP-02K07Q, Teledyne DALSA) whose maximal frame rate is 70 K frames per second. The setup’s physical photo is shown in Figure 1b.

The pixel arrangement of the polarization line scan CCD is showed as the inserted image in Figure 1a. The CCD consists of 4 rows, and each row has 2048 pixels. The upper three rows are linear polarizers with polarization angles of 0°, 135°, and 90°, respectively, and the last row is unfiltered. In this work, the upper three rows detect the linear polarization properties of the light sheet and record in the data acquisition card for further analysis.

Different from the two-dimensional CCD used in the imaging system or the photomultiplier used in the single angle or discrete angles, the polarization line scan CCD is used in the setup, which has 4 × 2048 pixels with a 12-bit depth; its acquisition speed is 70 kHz at maximum, which is quite different from the acquisition speed of the two-dimensional CCD (tens or hundreds Hz) or the photomultiplier (millions Hz). Because the individual particle passes the scattering volume within several microseconds [20], the polarization line scan CCD is suitable to effectively acquire the temporal signal of the light scattered by the individual particle. Meanwhile, considering the very weak intensity scattered by the individual particle in water, the high sensitivity is always needed, which is easy for the photomultiplier but is rather costly for the two-dimensional CCD. Due to the cylindrical lens in the setup, the scattered light is focused onto a light sheet to effectively enhance the intensity in different scattering angles. This design makes it possible for the angular distribution of the scattered light of individual particles to be acquired by the polarization line scan CCD, whose sensitivity is evaluated by the noise equivalent exposure as 9.8 $\mathrm{pJ}/{\mathrm{cm}}^{2}$.

### 2.3. Calibration

The intensities after three polarizers with different polarization angles are $I_{0}$, $I_{135}$, and $I_{90}$. The polarization state of light is known to be always represented by the Stokes vector, ***S***, whose linear part, $S^{L}$, is calculated by Equation (1).

(1) $$S^{L}=\left[\begin{array}{c} I\\ Q\\ U \end{array}\right]=\left[\begin{array}{c} I_{0}+I_{90}\\ I_{0}-I_{90}\\ I_{0}+I_{90}-2I_{135} \end{array}\right]$$

where *I* is the total intensity, *Q* and *U* are, respectively, the residual intensities at linear polarization angles 0° and 45°. Subsequently, *Q* and *U* are normalized by *I* to obtain polarization parameters *q* (≡*Q*/*I*) and *u* (≡*U*/*I*), respectively, which makes both the *q* and *u* range from −1 to 1.

The pixels of polarization line scan CCD may have some production deviations in the polarization angle, extinction ratio, and intensity response [27]. To reduce the measurement error caused by production deviations, the CCD pixels are first calibrated. Before the calibration, a 20-mm-diameter uniform light beam is modulated by rotating a polarizer with equally spaced interval from 0° to 170°, to obtain 18 polarization states of the light, and their polarization states, $S_{ref}$, are measured separately by a commercial polarimeter (PAX1000IR1, Thorlabs, Newton, NJ, USA). The linear parts of these Stokes vectors are then combined into a matrix as ${\left[S_{ref}^{L}\right]}_{3\times 18}$. Each modulated light beam is measured by CCD and each polarization state is calculated by Equation (1), and they are combined into a matrix as ${\left[S_{meas}^{L}\right]}_{3\times 18}$. Subsequently, calibration matrix A for each column of the pixels in CCD can be calculated by solving Equation (2), where *pinv*(*) presents the pseudoinverse calculator for a matrix.

(2) $$A={\left[S_{meas}^{L}\right]}_{3\times 18}\times pinv\left({\left[S_{ref}^{L}\right]}_{3\times 18}\right)$$

To testify the polarization calibration of the CCD, we illuminate it with a uniform parallel light beam with a 10° linear polarization state. To quantitatively evaluate the accuracy of the polarization detection, we calculate the degree of linear polarization (*DoLP*) and the angle of polarization (*AoP*) to evaluate the effects of the calibration, and they are defined as Equations (3) and (4).

(3) $$\;\;DoLP=\;\frac{\sqrt{Q^{2}+U^{2}}}{I}\;\;\;\;$$

(4) $$\;\;AoP=\left(1/2\right)\tan^{-1}\left(U/Q\right)$$

**Figure 2:**
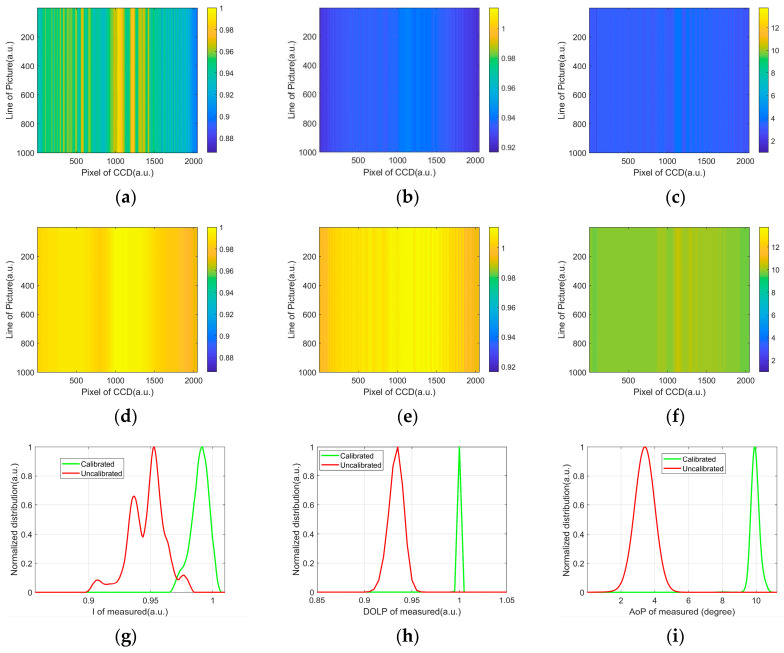
Uncalibrated (**a**–**c**) and calibrated (**d**–**f**) results of the scattered intensity *I*, *DoLP*, and *AoP*, and their normalized histograms (**e**–**i**).

The uncalibrated and calibrated results are shown in the first two rows of Figure 2, where the vertical axis of the images is 1000 times the measurement. The uncalibrated and calibrated results of the intensity are showed in Figure 2a,d, and the two intensity images are normalized to the maximum in Figure 2a. One can see that Figure 2d looks much more uniform than Figure 2a. The histograms of Figure 2a,d are shown in Figure 2g, which indicates that the calibration improves the intensity measurement. Meanwhile, the images of uncalibrated and calibrated *DoLP* are shown in Figure 2b,e, respectively. Figure 2e seems much more uniform than Figure 2b, even though their values are quite different, which can be explicitly described by their histograms shown as Figure 2h. One can see that the distribution of calibrated *DoLP* is much narrower than those of the uncalibrated *DoLP* in Figure 2h. Similar results about the uncalibrated and calibrated *AoP*, and their histogram, are shown in Figure 2b,e,I, respectively. Except for the uniformity, the calibrated values are closer to the true values than the uncalibrated ones; the true value is 1 for *DoLP* and 10° for *AoP*, respectively. The results in Figure 2 indicates that the CCD after calibration can effectively measure the linear part of the polarization state of the incident light.

### 2.4. Signal Processing

Because PH in Figure 1 is placed on the focus of L3 in the receiving path, the location and size of the detection volume in the sample pool are determined. The intersectional part of the detection volume and the light spot in the illuminating path is the scattering volume. Considering the numerical aperture of L1 and L2, and the size of PH (0.5 mm), the effective probing volume of the setup is considered to be the same as the scattering volume, which is less than 0.1 μL, and the equivalent field of view is less than 0.2 square millimeters. So, if the concentration of the suspended particles is less than 10^4^ particles per mL, there is at most only one particle in the scattering volume, which ensures the individual particle measurement. When the particle passes through the scattering volume, the scattered light contributes to a temporal pulse as the signal, and, generally, there is only quite low background originated from the scattering of water or the environmental light through PH.

To test the ability of the experiment setup, we prepare an aquatic suspension of SiO_2_. During the experiments, the scattering signals are quickly and continuously recorded by the data acquisition card (Xtium-CL MX4, Teledyne DALSA, Waterloo, ON, Canada) at a sampling rate of 20 kHz, and a series of temporal pulses can be obtained. Therefore, the polarization parameters, *I* =I(n,t), *q* =q(n,t), and *u* =u(n,t) are calculated, where *n* = 1, 2, …, 2048.

**Figure 3:**
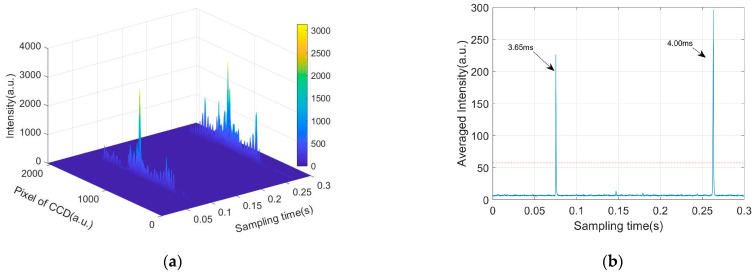
(**a**) Scattered intensity with pixel of CCD and sampling time. (**b**) Temporal signal of averaged intensity $\bar{I}\left(t\right)$; red dotted line: threshold to select pulses.

Figure 3a shows a piece of the signal of the measured parameter *I*, which are both indexed to the sampling time and the pixels of the CCD. Obviously, one can see the two pulses along the sampling time, which span along the pixels. The temporal pulses originate from the optical scattering of the suspended particles passing through the scattering volume. However, note that the spanning of the pulses does not cover the full pixel range of the CCD. To facilitate our future analysis, we only consider the middle 1458 pixels that fully cover the effective signals and re-index them from 1 to 1458.

To find the temporal locations of the pulses, the averaged intensity is obtained at $\bar{I}\left(t\right)\equiv \overset{1458}{\underset{i=1}{\sum }}I\left(i,t\right)/1458$. $\bar{I}\left(t\right)$ is shown in Figure 3b, and the red dotted line is the threshold, which is 8 times larger than the background. By using the threshold, the pulses can be found, their peaks are used to determine the temporal locations, and the widths around 4 milliseconds (ms) in Figure 3b describe how long the individual particles pass through the scattering volume. From Figure 3, the pulses with peaks lower than the threshold are omitted in order to ensure the high quality of the signals. Until now, a pulse set can be obtained and each pulse is a temporal slice, that is, $\left\{{\bar{I}}_{i}\left(t_{i}\right),t_{i}\in \left[T_{i,1},T_{i,2}\right],i=1,2,\ldots ,k\right\}$, where *k* is the total number of pulses and $T_{i,1},T_{i,2}$ are, respectively, the starting and ending time of the *i* th pulse.

For the *i* th pulse, we have the signal $I_{i}\left(n,t_{i}\right)$, where n=1,2,…,1458. To simplify the processing procedure and reduce the noises, the temporal data is averaged to $\tilde{I_{i}}\left(n\right)=\overset{T_{i,2}}{\underset{T_{i,1}}{\sum }}I_{i}\left(n,t_{i}\right)dt_{i}/\left(T_{i,2}-T_{i,1}\right)$. Because CCD acquires the pixels simultaneously, *q* and *u* share the same temporal slice as *I*, then $\tilde{q_{i}}\left(n\right)$ and $\tilde{u_{i}}\left(n\right)$ can be obtained similarly with $\tilde{I_{i}}\left(n\right)$.

## 3. Results

### 3.1. Comparison of Measured PCLAR with Those Simulated by Mie Theory

Because the pixel location corresponds to the scattering angle of the scattered light, experiments on the SiO_2_ suspension are conducted to derive the detecting angular range of our measured scattered light and verify the feasibility and accuracy of our experiment setup. Mie theory is a classical algorithm for analyzing the interaction between a spherical scatterer and light [28]. Note that micron-size particles in water have a close diameter with the wavelength of the light source, so Mie theory can be applied to analyze the theoretical distribution of the scattering intensity at different angles. We first measure the silica microsphere individually by our experiment setup, and then the measured PCLAR are compared with the result of the Mie theory.

**Figure 4:**
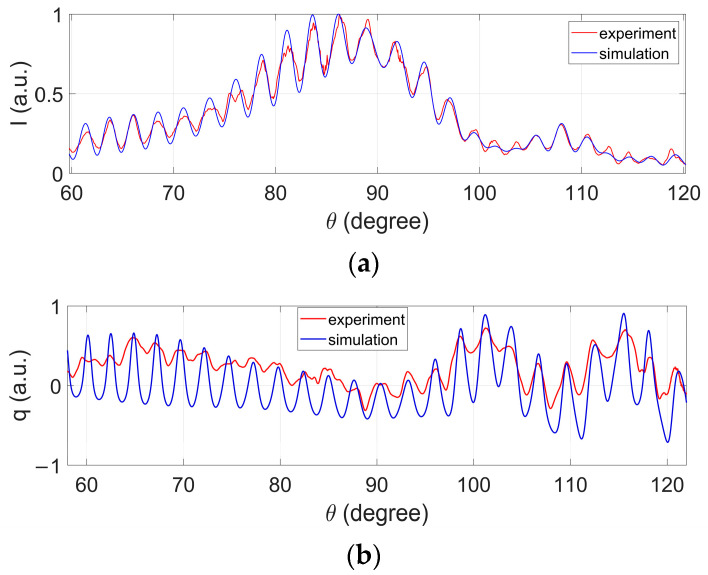

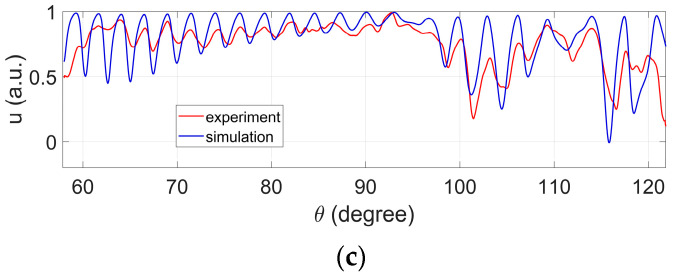
Experiment and simulated results of scattered intensity *I* (**a**), polarization parameters *q* (**b**), and *u* (**c**) for SiO_2_.

In the simulation, the refractive index of SiO_2_ is set as 1.451 + 0.0030i with a diameter of 10 μm, while that of water is 1.330. Figure 4 shows the measured and simulated results, which indicates that the experimental results are well consistent with the simulated results. In Figure 4a, the intensity distributions are normalized to their own maxima, and for all distributions, the locations of the peaks and the valleys for the measured and simulated results match well. The dynamic range between the peaks and valleys of the measured results seems smaller than those from the simulation, especially for the polarization parameters, which may result from the temporal averaging within the pulse when calculating $\tilde{q_{i}}\left(n\right)$ and $\tilde{u_{i}}\left(n\right)$. By comparing the measured and simulated results, $\tilde{q_{i}}\left(n\right)$ and $\tilde{u_{i}}\left(n\right)$ are accordingly considered as $q_{i}\left(\theta \right)$ and $u_{i}\left(\theta \right)$, and the angular range covers the range from 60° to 120°. Until now, $q_{i}\left(\theta \right)$ and $u_{i}\left(\theta \right)$ are the so-called PCLAR. Note that the angle range are divided into 1458 pixels, and generally the angular resolution is around 0.08° per pixel, which ensures a high angular resolution. In the following context, both the polarization parameters, *q* and *u* are the 1 × 1458 vectors.

### 3.2. Classification of Six Categories of Particles

**Figure 5:**
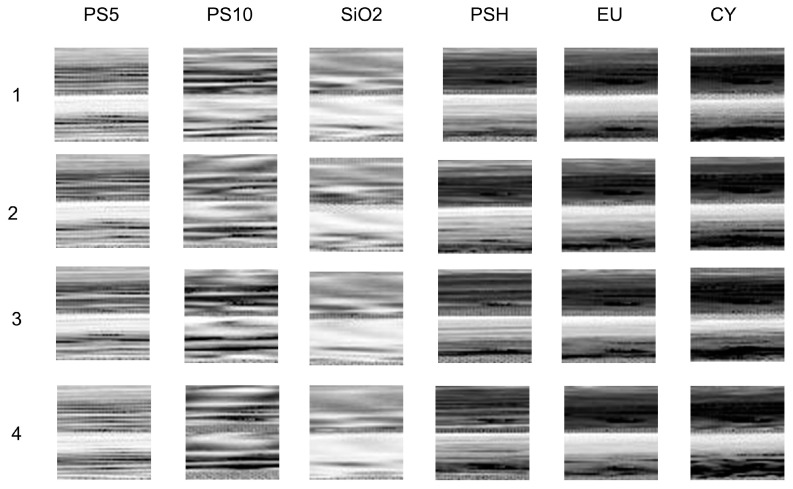
Grayscale maps realigned from PCLAR for six categories of particles.

In the aquatic environment, different categories of particle are undoubtedly mixed, and the particulate composition information is always important for environmental monitoring [29]. Before that, we first investigate the classification ability of PCLAR. Different samples in Section 2.1 are measured separately and fed to the convolutional neural network (CNN) to build the classifier. In our work, we focus on the polarization parameters *q* and *u*. To fit into the input of CNN and take full use of these parameters, we combine them as a 1 × 2916 vector [*q*, *u*], and then realign them into a 54 × 54 image. Every realigned image corresponds to one particle. The grayscale images of six categories of particles are shown as Figure 5, and the stripes and speckles in the images correspond to the angular vibrations of the polarization parameters. The distributions in the columns of Figure 5 seem to be robust for each category, but those along the rows for different categories are quite different.

**Figure 6:**
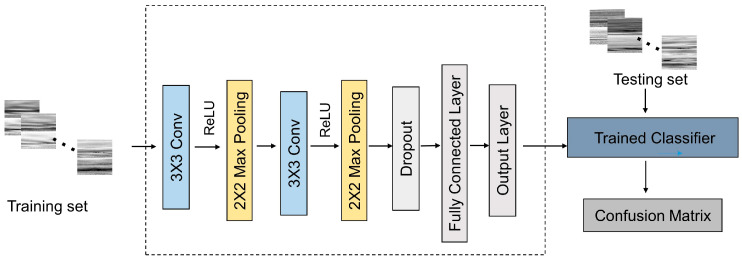
Flowchart of classification process.

The images are then processed by the CNN algorithm, a well-known tool for image classification tasks, and the architecture in this work is shown as the dashed box in Figure 6. For each category, 1000 images are measured to form its dataset, where 800 images form the training set, and the left 200 images form the testing set. Then, 4800 images are used to train the classifier, and the other 1200 images are used to test the classifier. Finally, the performance of the classifier is quantitatively evaluated.

**Figure 7:**
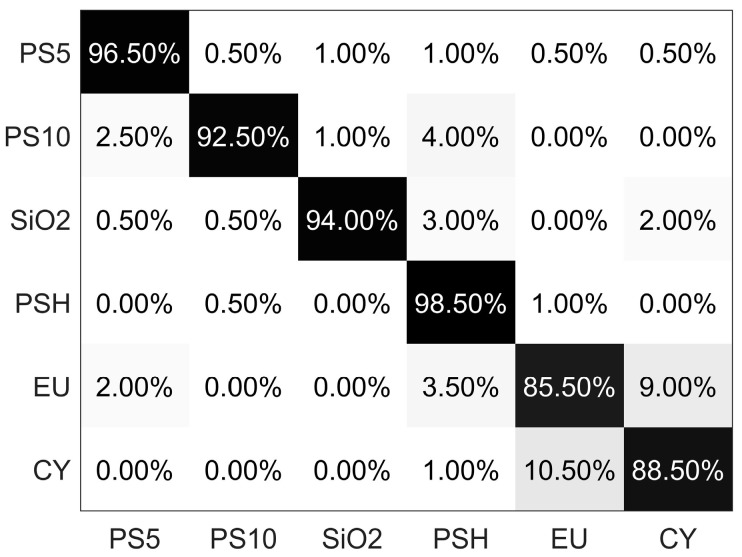
Confusion matrix of the classifier on the testing dataset.

The performance of the classifier is visualized by the confusion matrix, as shown in Figure 7. The result shows that all the accuracy values are larger than 85%, and the overall accuracy is more than 90%, which demonstrates that all the categories can be well classified from each other based on PCLAR. The current 6 categories of particles cover the different physical properties, such as the size, shape, structure, and refractive index, and the convincing results in Figure 7 demonstrate that PCLAR can effectively describe the optical difference originated from the physical properties. Additionally, we notice that the classification accuracy suffers from the mutual confusion between the microalgae EU and CY. Obviously, there are more abundant data in PCLAR than the discrete angles detection methods or the intensity-only detection methods. Besides that, the large angular range and the polarization measurement give PCLAR more opportunity to include specific information that is sensitive to the physical properties.

### 3.3. Identifying the Particulate Compositions in Mixtures

Based on the excellent classification ability of PCLAR with the help of CNN, we subsequently try to identify the particulate compositions in mixtures, which is desired for in situ environmental monitoring [30]. The mixture experiments of the silica microspheres and microalgae CY are then conducted.

Firstly, 50 μL of SiO_2_ concentrate is added into 5 mL distilled water in the sample pool (group 1) and is then measured by the setup. Then, 50 μL CY is sampled from its concentrate and successively added into the sample pool for three rounds (group 2~4), and finally 100 μL CY is added for the fourth round (group 5). After each addition, the sample is measured for 100 *s* to obtain PCLAR; the stirrer rotates at a speed of 150 rpm to keep these particles suspended in the sample pool.

**Figure 8:**
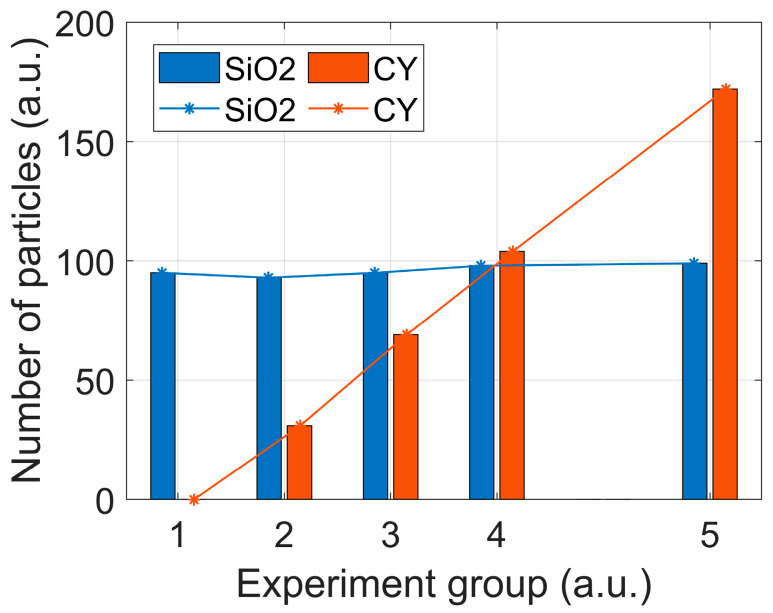
Group bar plot for particle number of SiO_2_ and CY. Groups 1~5 corresponds to mixtures with total volumes of CY concentrate, 0, 50, 100, 150, and 250 μL, respectively.

A new CNN classifier based on the above separate measurements is built to classify SiO_2_ and CY, whose classification accuracy is larger than 95%. The results are shown in Figure 8, and the numbers of SiO_2_ weakly fluctuate around the average, but those of CY are proportional to the adding volume of the concentrate. Moreover, the concentration proportion between SiO_2_ and CY in the mixture can be easily retrieved. The individual particle measurement and PCLAR empower the setup to classify the particulate composition and their concentration proportion in the mixtures. This ability enables this method to be instrumented when in situ probing the particles in water.

## 4. Discussion

### 4.1. Performance Comparisons of Different Anglular Selectin Strategies

This method is designed to rapidly measure the scattered polarization parameters of the individual suspended particle from 60° to 120°, whose power is demonstrated by the excellent classification result of different categories of particles. To further show the contribution of different angles quantitatively, we compare the classification performance of the scattered polarization measurement with other angular selection strategies, i.e., the single angle (120°), discrete angles (60°, 90°, 120°), forward continuous angles (from 60° to 90°), and backward continuous angles (from 90° to 120°). The data with these angular selection strategies are fed to the CNN model to train and test, and they are the same as in Figure 6.

**Figure 9:**
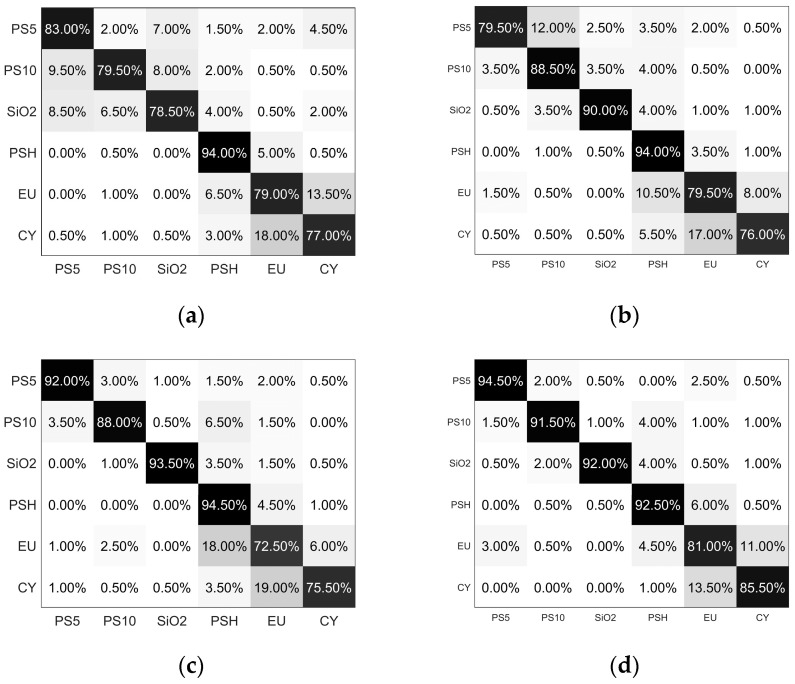
Confusion matrixes for different angular selection strategies. (**a**) single angle: 120°; (**b**) discrete angles: 60°, 90°, 120°; (**c**) forward continuous angles range from 60° to 90°; (**d**) backward continuous angles range from 90° to 120°.

The confusion matrixes of the classifications are shown in Figure 9. From the confusion matrixes, the recognition accuracy based on the single angle (Figure 9a) for PS10 and SiO_2_ are the lowest, and the recognition accuracy of PS5, EU, and CY are at lower levels than those with other angular selection strategies. This is understandable due to the limited information included in this single angle. Because only the linear polarization parameters are considered, the recognition accuracy at 120° is lower than the previous works [31]. For the strategy of discrete angels, as shown in Figure 9b, the recognition accuracy of PS10 and SiO_2_ are improved, which means the scattering angles of 60° and 90° introduce some specific information for PS10 and SiO_2_. Meanwhile, the recognition accuracies of the other categories do not benefit from the additional angles, which means adding information from 60° and 90° contributes nothing to the classification. We should be aware of the importance of the selection of the discrete angles. However, even though the discrete angles are sophisticatedly selected in the literatures, their parameters still exclusively work for limited cases [32].

For the forward continuous angles in Figure 9c, the recognition accuracies of PS5 and SiO_2_ become better than those with the single angle and the discrete angles, but those of EU and CY become worse. The latter case may be explained by the absence of scattered polarization parameters at 120°, which has been proven to be essential for particle discrimination [20]. For the backward continuous angles in Figure 9d, all the particles except for SiO_2_ and PSH can be more accurately recognized than the other three cases in Figure 9. Note that the recognition accuracies of SiO_2_ and PSH are also larger than 92%, and the lower accuracies of SiO_2_ and PSH in Figure 9d compared to Figure 9c means that there is some particular information included in the forward angles.

It is obviously that the recognition accuracies of all categories in Figure 5 are larger than those in Figure 9. We further compare the overall classification accuracy for all the angular selection strategies, which is shown in Table 1. One can see that the recognition accuracy of the angular coverage from 60° to 120° is the best, those from 90° to 120° are the second best, and the single angle is the worst. Thus, the angular selection strategy is important for particle recognition, which needs to be paid much attention to for the scatterometer. It is easily understandable for the best performance of PCLAR becuse they continuously cover the largest angular ranges and benefit from them. It should be noted that, in our method, PCLAR is acquired at a single shot and can be rapidly measured at 20 kHz, which does not suffer from the continuously larger angular range.

### 4.2. Simulated PCLAR of the Four Non-Biological Microspheres

**Figure 10:**
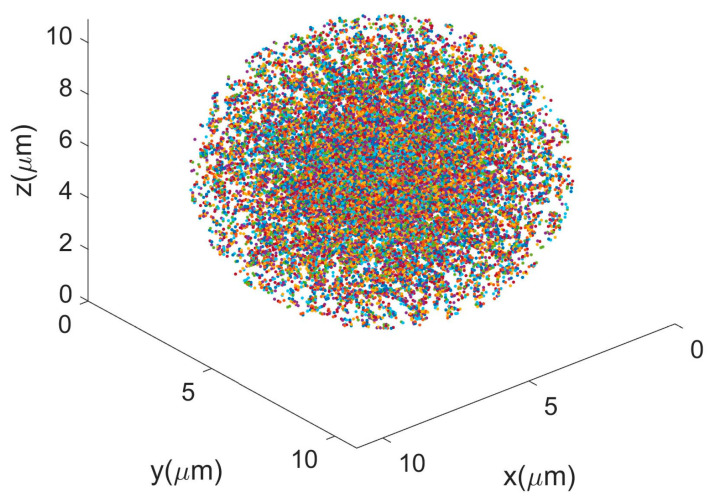
Geometric model of PSH for DDA simulation.

Section 3.2 shows that four categories of non-biological microspheres (PS5, PS10, PSH, SiO_2_) can be well differentiated from each other. Among these four, PS5, PS10, and SiO_2_ are uniform spheres, and Mie theory can be used to compute the scattered polarization parameters [33]. Because PSH is not uniform, it is simulated by the discrete dipole approximation (DDA) [34]. Specifically, in order to build the geometric model of PSH according to its classic structure [35], we first generate a smooth PS microsphere with 10 μm diameter, and then subtract the intersectional volume with the 200 small spheres whose central points randomly locate at the surface of the 10 μm diameter microsphere. The small sphere’s diameter is 0.4 μm. Here, we acquire the first version of the model. We then repeat the operation to subtract the intersectional volume with another 200 small spheres whose central points randomly locate at the surface of the last version of the model. Totally, we iterate this operation 50 times, and acquire the PSH’s geometric model, as shown in Figure 10. Then, we discretize the model and use DDA to calculate the angular distributions for the scattered polarization parameters.

**Figure 11:**
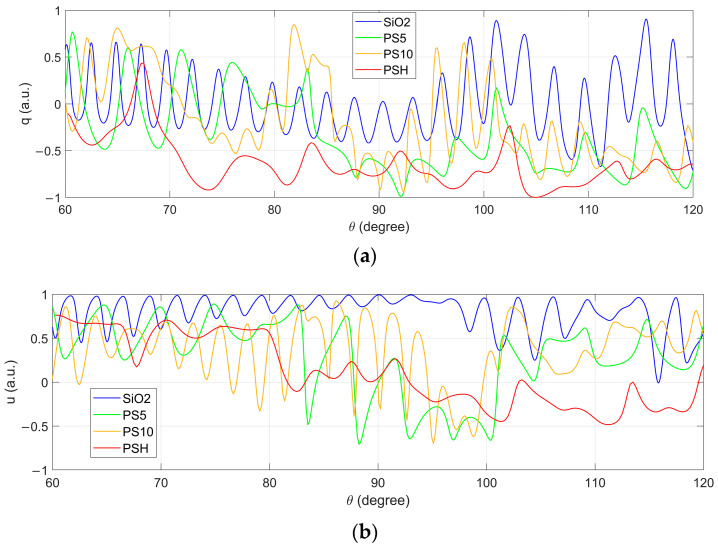
Simulated polarization parameters *q* (**a**) and *u* (**b**) of SiO_2_, PS5, PS10, and PSH.

Subsequently, the distributions of the polarization parameters *q* and *u* of these four categories of microsphere are collected, and these are shown in Figure 11. It can be seen that the peak-valley structure is all curves, while that of PSH has less vibrations than the other particles, which originates from the heterogeneous structure. Comparing the distributions of PS5 and PS10, fewer peaks and valleys can be observed in the distributions of the former compared to those of the latter, which originates from the size. Although the number of peaks and valleys of PS10 and SiO_2_ are similar, their locations are different in *q* and values are different in *u*. Generally, the *q* and *u* distributions of PSH look the most different to those of the others in both the vibrations and the values, which ensures the best recognition of PSH in all classifications in Figure 5 and Figure 9.

It should be emphasized that the difference between the particles exist in not only both the polarization parameters *q* and *u* but also in the different angles. For example, the *u* values are most different in the angles around 90° for SiO_2_ and PS10, but those for PS10 and PSH are in an angle near 120°. Relatively, there are more difference in *u* than in *q*, for all particles. These theoretical results demonstrate the measurement necessity of the scattered polarization parameters with continuously large angular range.

### 4.3. Effective Size and Refractive Index of Cells Retrieved from PCLAR

**Figure 12:**
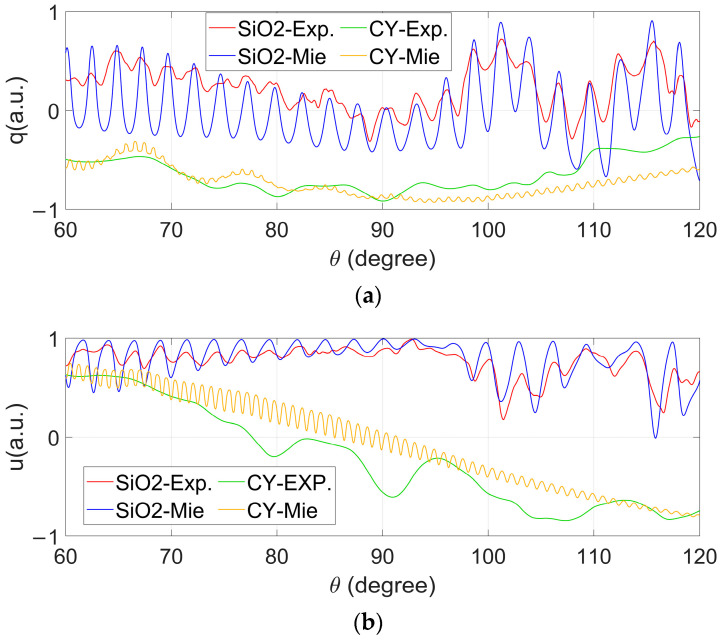
Polarization parameters *q* (**a**) and *u* (**b**). Experiment results of SiO_2_ (red) and CY (green); Mie theory simulation results of SiO_2_ (blue) and CY (yellow).

Taking advantage of the abundant and meaningful information carried by PCLAR, the further investigation of its potential to explicitly retrieve the particle’s physical properties is conducted below. The measured PCLAR of SiO_2_ and CY are shown in Figure 12 as the red and green lines. For both *q* and *u*, CY and SiO_2_ can be easily differentiated. The simulated PCLAR of SiO_2_ based on Mie theory shown in Figure 3 are recalled and shown in Figure 12 as the blue lines. Similar simulations are carried out for CY, and the solid sphere mostly fitted to CY’s measured PCLAR is acquired with a 30 μm diameter and 1.359 + 0.0045i refractive index, which falls in the ranges given in the literature [36]. The yellow lines in Figure 12 show the simulated PCLAR, which do not perfectly match with the experimental results shown as the green lines. Note that the shape of CY is stump-like, far from the sphere, and in the simulation, the size dominates the number and locations of the peaks in the angular distributions of *q* and *u*, but the refractive index is more sensitive to their value ranges than the size. The imaginary part of the refractive index of SiO_2_ is comparable with that of CY, which may originate from the residual polymer components in the silica microspheres during manufacturing [37].

## 5. Conclusions

In this paper, the methodological concept for measuring polarization parameters with a continuously large angular range, i.e., PCLAR, of individual suspended particle is proposed. The experimental setup is built to measure the scattered polarization parameters from 60° to 120° of the individual particles at single shot, and the measurement can be repeated at 20 kHz. The setup is validated by the suspension of silica microspheres. Experimental results show that PCLAR can well characterize the six categories of particles with different size, shape, refractive index, and structure, which enables the excellent performance of the classification equipped by CNN. Furthermore, PCLAR helps to effectively probe the particulate compositions in the mixtures of SiO_2_ and CY with different proportions. The comparisons with different angular selection strategies show that the continuously large angular range of PCLAR enables the best classification. Simulations of PCLAR for particles with different size, refractive index, and structure reveal the measurement necessity of both polarization parameters and the continuously large angular range, for the excellent discrimination of these particles. Moreover, with Mie theory, physical information such as the effective size and refractive index of individual microalgal cells can be retrieved. In summary, the method’s power, originating from the intrinsic advantages of PCLAR, are convincingly demonstrated in this paper. A future prototype based on this concept may provide a promising tool for particulate monitoring in aquatic environment.

## Figures and Tables

**Table 1:** Comparisons of the overall accuracies.

Angular Selection Strategy	Single Angle: 120°	Discrete Angles: 60°, 90°, 120°	Forward Continuous Angles, Range from 60° to 90°	Backward Continuous Angles, Range from 90° to 120°	Continuous Angles, Range from 60° to 120°
Overall accuracy	81.83%	84.58%	85.91%	89.50%	92.58%
